# Patient-derived podocyte spheroids reveal new insights into the etiopathogenesis of Alport syndrome

**DOI:** 10.3389/fcell.2023.1111424

**Published:** 2023-03-02

**Authors:** Ricardo Romero-Guevara, Orthodoxia Nicolaou, Benedetta Petracca, Sadr Shaheed, Christopher Sutton, Eleni Frangou, Marina Afami, Kyriacos Kyriacou, Adonis Ioannides, Christodoulos Xinaris

**Affiliations:** ^1^ Department of Basic and Clinical Sciences, University of Nicosia Medical School, Nicosia, Cyprus; ^2^ Department of Cancer Genetics, Therapeutics, and Ultrastructural Pathology, Cyprus Institute of Neurology and Genetics, Nicosia, Cyprus; ^3^ Laboratory of Organ Regeneration, Department of Molecular Medicine, Institute of Pharmacological Research “Mario Negri”, Bergamo, Italy; ^4^ Nuffield Department of Surgical Sciences, University of Oxford, Oxford, United Kingdom; ^5^ School of Chemistry and Bioscience, University of Bradford, Bradford, United Kingdom; ^6^ Department of Nephrology, Limassol General Hospital, Nicosia, Cyprus

**Keywords:** Alport syndrome, induced pluripotent stem cells, spheroids, disease modeling, podocytes

## Abstract

Alport syndrome (AS) is a rare disease characterized by defective glomerular basement membranes, caused by mutations in COL4A3, COL4A4, and COL4A5, which synthesize collagen type IV. Patients present with progressive proteinuria, hematuria and podocyte loss. There is currently no cure for Alport syndrome, and this is mainly due to its complex and variable pathogenesis, as well as the lack of models that can faithfully mimic the human phenotype. Here we have developed a novel human culture model of Alport syndrome and used it to study the effects of different mutations on podocyte development and biology. First, we established a differentiation protocol that allowed us to generate podocyte spheroids from patient-derived human induced pluripotent stem cells (hiPSCs). We have then carried out discovery proteomics and demonstrated that a total of 178 proteins were differentially expressed between Alport (AS1 and AS3) and control (LT) podocytes. GO analysis indicated alterations in several metabolic pathways, such as oxidative phosphorylation, RNA maturation, chromatin condensation, and proliferation. Although functional assays showed no changes in lactate production and mitochondrial potential compared to healthy controls, immunofluorescence and electron microscopy analysis showed key morphological changes related to the phenotypical maturation of Alport podocytes. Moreover, the studied mutations led to persistent proliferation, increased reactive oxygen species (ROS) production and the concomitant expression of peroxisome proliferator-activated receptors α and γ (PPARα and PPARγ) in podocytes. These data on patient-derived podocytes provide evidence that collagen mutations, in addition to playing a central role in the defective development of the glomerular filtration barrier, cause significant alterations in podocyte development and metabolism very early in development, even before the formation of the filtering apparatus. In conclusion, our study provides a new methodological platform for the differentiation of podocytes and to study human podocytopathies in a personalized manner, and reveals new insights into the etiopathogenesis and pathobiology of Alport syndrome.

## 1 Introduction

Alport syndrome (AS) is a genetic condition characterized by kidney disease, hearing loss, and eye abnormalities. Patients present with albuminuria and hematuria starting in childhood and progress rapidly to end stage renal disease (ESRD) before the fourth decade of life. AS is caused by mutations in *COL4A3*, *COL4A4,* and *COL4A5*, which encode for type IV collagen. This forms the mesh of the glomerular basement membrane (GBM), the central, non-cellular layer of the glomerular filtration barrier that is situated between two cellular components, the fenestrated endothelial cells and podocytes. At the structural level, the GBM in AS patients exhibits progressive deterioration, characterized by thickened and thinned areas of lamellar appearance, which affect the filtration capacity of the glomeruli. During the course of the disease, podocytes undergo effacement, detachment and cell death, crucially determining disease progression. Save the angiotensin converting enzyme (ACE) inhibitors that delay disease progression in some cases, there are currently no specific treatments for AS.

The assembly of the GBM is a complex developmental process in which α1 α1 α2 type IV collagen chains assemble into trimers intracellularly and are then secreted by podocytes and endothelial cells in the developing glomeruli. Later, the fetal GBM is replaced by the mature version of it, composed of α3 α4 α 5 type IV collagen chains, which are secreted primarily by podocytes. This shift in expression of α chains does not occur in AS, meaning that the adult GBM in these patients is immature and not ready for the strain forces that adult glomeruli have to face. Therefore, although the disease is considered to be caused by physical changes in the GBM that affect the podocyte and glomerular filtration, recent evidence points toward a direct biological effect played by collagens through cell membrane receptors (Yeh et al., 2012; Kim et al., 2020).

Some of the most important limitations on studying the mechanisms underlying AS pathophysiology and developing efficient therapeutic approaches are the lack of models that can realistically mimic the human phenotype, the complex pathobiology of the disease, and the high phenotypical variability between patients. Here we set to develop an *in vitro* model that can be efficiently used to study human AS in a personalized manner. To this end, we have first optimized a protocol for the generation of 3D podocyte spheroids from human induced pluripotent stem cells (hiPSCs) (Takahashi et al., 2007) that were obtained from AS patients. We then used this methodology to identify new molecular pathways affected by AS-causing mutations, highlighting the utility of our system for studying AS and potentially other podocytopathies, and identifying pathways for future pharmacological intervention.

## 2 Methods

### 2.1 Cell culture

Alport disease-derived human induced pluripotent stem cells—AS FiPS 1-Ep6F-2 (AS1) and AS FiPS 3-Ep6F-9 (AS3)—were obtained from the Barcelona Stem Cell Bank (CMRB) (Kuebler et al., 2017a; Kuebler et al., 2017b). The control episomal hiPS cell line (Thermo Fisher #A18945) was used for comparison. hiPSCs were kept undifferentiated using Essential 8 flex media (Thermo Fisher) on geltrex (Thermo Fisher)-coated cell culture plasticware. Media was changed every day, or every other day in low density cultures.

For differentiation experiments, DMEM/F12 media, Neurobasal media, non-essential amino acids (NEAA), N2 supplement, B27 supplement, Insulin-Transferrin-Selenium (ITS), Accutase and phosphate buffer solution (PBS) were obtained from Thermo Fisher. Rock inhibitor Y-27632, ethylenediaminetetraacetic acid (EDTA) and retinoic acid (RA) were purchased from Sigma Aldrich. Bone morphogenic protein 4 and 9 (BMP4 and BMP9) and fibroblast growth factor 9 (FGF9) were obtained from Peprotech. 3-(3-amino-phenyl)-4-(1-methyl-1H-indol-3-yl)-pyrrole-2,5-dione, referred to as CP21R7, 4-[(3aR,4S,7R,7aS)-1,3,3a,4,7,7a-hexahydro-1,3-dioxo-4,7-methano-2H-isoindol-2-yl]-N-8-quinolinyl-benzamide referred as IWR1 and 6-[[2-[[4-(2,4-dichlorophenyl)-5-(5-methyl-1H-imidazol-2-yl)-2-pyrimidinyl]amino]ethyl]amino]-3-pyridinecarbonitrile known as CHIR99021 and 4-[4-(1,3-benzodioxol-5-yl)-5-(2-pyridinyl)-1H-imidazol-2-yl]-benzamide, hydrate referred to as SB431542 were obtained from Cayman chemicals. Apel 2 media was purchased from Stem cell technologies.

On the day of seeding (day 0) undifferentiated hiPSC colonies were rinsed with PBS following 7 min of incubation in a 0.5 mM EDTA/PBS solution at room temperature for cell detachment and harvesting. Plates were rinsed with an additional 1 mL of DMEM/F12 media and the cell suspension was collected in a falcon tube. The single-cell suspension was centrifuged for 5 min at 700 g. The pellet was resuspended in 1–2 mL of Essential 8 flex media and 10 µM rock inhibitor Y-27632 for single-cell survival. Cells were then seeded on the same media formulation on geltrex-coated plates at a density of 30,000 cells/cm^2^ in 6 well plates. The following day (day 1) media was changed to induction media, composed of 1 DMEM/F12: 1 Neurobasal media with 1X N2, 1X B27 supplements, 1 μM C21R7, 25 ng/mL BMP4 and media was changed on day 3 with the same media formulation. On day 4, media was changed to day 4 media (Apel 2 media), 100 nM RA, 50 ng/mL BMP7, 200 ng/mL FGF9, using 2 mL per well in a 6 well plate. On day 6, medium was removed, cells were rinsed with 1 mL PBS, and harvested with 1 mL/well of Accutase for 5–10 min and resuspended in a falcon tube with an additional 3 mL of DMEM/F12 media. Cells were counted, centrifuged and seeded in 35 mm Petri dishes without cell culture treatment at a density of 1-3 million cells per dish in day 6 media (DMEM/F12, 1X N2, 1X ITS, 1X NEAA, 3 µM CHIR99021, and 10 µM Y27632). The next day (day 7) cells were harvested and dissociated mechanically with the help of the pipette, counted, centrifuged and resuspended in day 7 media (DMEM/F12 mix, 1X N2, 1X ITS, 1X NEAA, 2 µM IWR1, 10 μM RA, 5 μM SB431542 and 10 ng/mL FGF9) and seeded in cell culture inserts at 800,000 cells/insert in 100 µL of day 7 media, and 600 µL of day 7 media in the basolateral compartment. Medium was changed from the basolateral compartment on day 10 and day 13.

### 2.2 Immunofluorescence

Cells that had grown on top of cell culture inserts were quickly blotted on absorbent paper (from the bottom), and 500 µL of paraformaldehyde (PFA) 3.7% were immediately added to each insert hold in a 12-well plate and incubated at room temperature for 20 min. PFA was gently removed with dry tissue, and inserts were cut from the plastic chamber and placed on top of glass microscope slides. Then each 200 µL/insert was washed twice with PBS for 5 min. For blocking, the inserts were incubated for 30 min at room temperature in blocking buffer (PBS, 0.1% triton X, 1% BSA, and 10% FBS). Following blocking, primary antibody was prepared according to the concentrations in Table 1, in the same blocking buffer w/o triton X, and cells were incubated in 70 µL/insert of primary antibody mix overnight at 4°C in a humidified chamber. Following overnight incubation, samples were washed three times with PBS at room temperature (5 min/each 200 µL/insert). Tissue paper was used between washes for drying. Secondary antibody was prepared in blocking buffer and 2 μg/mL Hoechst 33,258 (Cayman chemicals) were added and cells were incubated in this mix for 1 h at room temperature in the dark. Finally, after two washes in PBS for 5 min/each in the dark, the samples were air dried, treated with glass slowfade (Thermo Fisher) and covered with a glass coverslip.

**TABLE 1:** Antibodies and reagents used in immunofluorescence (IF) and western blot (WB) analysis.

Antibody target	Code	Details
*NPHS1*	sc-377246, Santacruz	Mouse monoclonal 1:100 IF
*WT1*	sc-7385, Santacruz	Mouse monoclonal 1:100 IF
*ATP5A*	sc-136178, Santacruz	Mouse monoclonal 1:100 IF
*NPHS2*	sc-518088, Santacruz	Mouse monoclonal 1:100 IF
*Goat anti-mouse Alexa 488*	A11029, Thermofisher	1:500 IF
*Hoechst 33,258*	16,756, Cayman	2 μg/mL IF
*ACTNB*	sc-47778, Santacruz	Mouse monoclonal, 1:2000 WB
*Mouse IgG Fc BP-HRP*	sc-525409, Santacruz	Mouse IgG Fc HRP conjugated, 1:1000 WB
*Phalloidin-Atto 488*	49409, Sigma	1:200 IF
*Ki-67*	ab16667, Abcam	Rabbit monoclonal 1:200 IF

### 2.3 Western blot (WB)

To harvest the cells, the medium in the basolateral compartment was aspirated while 400 µL of Accutase were added on top of the inserts (apical compartment) and incubated for 8–10 min. Then an additional 400 µL of DM/F12 media were added and with the help of the pipette cell clumps were disaggregated and collected in a 2 mL centrifuge tube. Another 600 µL of media were used to wash and collect any remaining cells in the inserts and the mix was spun down for 5 min at 1,500 g. Media supernatant was then carefully removed, and cell pellets were suspended in 50–100 µL of radioimmunoprecipitation assay (RIPA) buffer supplemented with protease inhibitor cocktail (Pierce). The lysis of the cell pellet was accomplished using the pipette tip first, followed by a 1 mL hypodermic needle. Protein was quantified using Bradford reagent (Sigma Aldrich) following the manufacturer’s instructions. Samples run in SDS electrophoresis gels and semidry transfer was done in activated PVDF membranes. Primary antibodies for WB were diluted in tris-buffered saline with 0.1% Tween 20 detergent (TBS/T), 5% skimmed dry milk at the concentration stated in Table 1 and incubated at 4°C overnight in a rocking plate. The next day, membranes were washed three times in TBS/T for 5 min each, and secondary antibody mix was added for 1 h at room temperature. An additional three washes in TBS/T were done before ECL Plus (Pierce) substrate was added and images were taken.

### 2.4 RNA extraction and Q-PCR

The cells were harvested with Accutase, as described before, for WB, with the only difference that cell pellets were resuspended in 500 µL of Trizol reagent for the RNA extraction following the manufacturer’s instructions (Thermo Fisher). RNA was resuspended in 20–60 µL of H_2_O, depending on the size of the visualized RNA pellet collected. RNA was quantified in nanodrop. 1.5 µg of RNA were used for retrotranscription in a total volume of 30 µL using the High-capacity cDNA reverse transcription kit (Thermo Fisher) that uses random primers for cDNA synthesis and following the manufacturer’s protocol. cDNA was then diluted 1:10 for Q-PCR. For Q-PCR 2X SYBR green PCR master mix (Thermo Fisher) and 10 µmol of each primer were mixed into a total volume of 20 µL per well and reactions were carried out in triplicate following the manufacturer’s instructions. The primers used in this work are shown in Table 2. Reaction conditions were as follows: 95°C 1°min, 40 X (95°C 30°s, 60° 30 s), 95°C 30°s, 72°C 5°min, followed by melting curve 50°C–95°C, +1°C/20 s. All of our primers displayed single peak in the melting curve analysis. For relative quantification analysis we followed the Δ-ΔCt method, using ACTNB as the reference gene and the control condition according to how it was defined in each experiment.

**TABLE 2:** Primers used for Q-PCR analysis.

Gene	Fwd	Rev
*NPHS1*	GAC​CAT​TGC​CAA​CGT​GTC​TG	CTTGCCACCGTTCATTCT
*SYNPO*	TCT​ACC​ATG​GCT​ACC​TGC​CT	TTC​CGG​GTA​GAG​AAG​GAG​GG
*MAFB*	TTG​TAA​CCA​GAA​TCA​CCC​TGA​GGT​C	CCA​GGG​TCA​GGG​ATG​GCT​AA
*WT1*	ACA​GAA​TAC​ACA​CGC​ACG​GT	GGC​GTT​TCT​CAC​TGG​TCT​CA
*PAX2*	AGG​CAT​CAG​AGC​ACA​TCA​AAT​CAG	TCA​GGG​TTG​GTG​GAT​GCA​GAT​A
*SIX2*	CTTGCCACCGTTCATTCT	GGACCAGGACACAGAGTA
*OSR1*	GAC​ATC​TGC​CAC​AAA​GCC​TTC	CCC​ACA​GGT​TCT​ATT​TAG​CAT​TTG​A
*LAMA1*	AGT​TTC​GAA​CCT​CCT​CGC​AG	CTG​TTA​TCC​TGC​CAG​CAC​CA
*LAMA2*	ATG​AAA​GCA​AGG​CCA​GAA​GT	CTC​CAG​GGA​ACA​TCC​TTT​GGT
*LAMB1*	CAA​TGA​GTT​CAC​GGG​GCA​GT	GTC​ACA​CTG​TGG​CGT​CTC​AA
*LAMB2*	CAC​CTC​CCC​TTA​TCC​CTG​TTC	GCC​AGC​ACG​CTT​AGC​AGT​AG
*COL4A1*	GAA​GGG​TGA​TCC​AGG​TGA​GA	CAC​CCT​TGT​CAC​CTT​TTG​GT
*COL4A2*	GCCCAGAGAGCCCAGCAAG	CAGTCCCACTTAGCCTCGG
*COL4A3*	TCCCAGGAAGACAAGGCGC	GGCACCTGGGAAACCTGGA
*COL4A4*	TGAAGGGAAATCCCGGTGTG	CAGGTGGCTCTACCAACAGG
*COL4A5*	GCCTGGGCTAAAGGGTCTAC	CAAACCACGGGTACCTGGC
*PPARA*	TGG​GAA​GGC​AGC​GTT​GAT​TA	CTG​GCA​GTT​CCA​GTC​CAG​AT
*PPARG*	GTG​CAG​CTA​CTG​CAG​GTG​A	TGG​CTC​AGG​ACT​CTC​TGC​TA
*TGFB1*	TTG​ACT​TCC​GCA​AGG​ACC​TC	CTC​CAA​ATG​TAG​GGG​CAG​GG
*EGF*	ACC​TCA​AGA​ATG​GGG​GTC​AAC	GCC​TCC​ATG​AAG​TTG​GTT​GC
*SOD1*	GGT​GTG​GCC​GAT​GTG​TCT​AT	CCT​TTG​CCC​AAG​TCA​TCT​GC
*SOD2*	CTG​GAA​GCC​ATC​AAA​CGT​GAC	GCC​TTG​GAC​ACC​AAC​AGA​TG
*ACTNB*	TGG​CAC​CCA​GCA​CAA​TGA​A	CTA​AGT​CAT​AGT​CCG​CCT​AGA​AGC​A

### 2.5 Metabolic assays

ATP quantification was performed by harvesting cells as described in the RNA extraction section, with the only difference that before centrifuge, the cell number in the suspension was quantified and at least 100.000 cells were taken for further analysis. Then cells were centrifuged at 15,000 g for 5 min, and the pellet was resuspended in boiling water and incubated at 98°C for 10 min before being quickly frozen for ATP quantification using the ATP quantification kit (Thermo Fisher) in the luminescence plate reader.

For Mitotracker analysis (Thermo Fisher), on day 14 podocytes were incubated with Accutase for 7–10 min and harvested as described above. After spinning down and resuspension in day 7 media (see cell culture methods), 150,000 cells were seeded per well in a 96 well plate in duplicate for each treatment and/or cell line in a 90 μL cell culture volume. Then, an additional 20 µL of DM/F12 or H_2_O_2_ (3 mM final) and 10 µL Mitotracker 400 nM were added and cells were incubated for 1 h (5% CO_2_, 37°C). Fluorescence microscopy was performed at 570 excitation and 600 emissions. Then fluorescence intensity was quantified and expressed per cell area. JC-1 analysis was performed following the manufacturer's instructions (Thermo Fisher).

For ROS analysis, the ROS assays kit (Sigma Aldrich) was used, following the manufacturer’s instructions. Briefly, cells were harvested as before and seeded at 150,000 cells/well in a 96 well plate in 90 µL volume in duplicate or triplicate, then the 100 µL ROS reaction detection mix and an additional 20 µL of DMEM or 3 mM H_2_O_2_ in DMEM were added to each well. Cells were incubated for 2 h in a CO_2_ incubator and fluorescence microscopy analysis was performed at 520 excitation 605 emission.

In both assays, a well with media and treatment(s) were used for reading correction in each experiment.

An L-Lactate assay kit (Sigma Aldrich) was used to measure glycolysis, according to the manufacturer’s instructions. On day 13, podocyte medium was changed and 4 h later an aliquot of 50 µL per insert was taken as a starting reference value, then at 24 h (day 14) a second aliquot was taken and cells were harvested and counted. Then, during L-Lactate measurement, a standard curve was set up and samples diluted 1:10, then 40 μL L-lactate reaction mix and 10 µL were mixed per well in a 96-well plate and two absorbance readings were taken at 565 nm, with 20 min of incubation time in between. The values were calibrated against the standard curve and the first reading following the manufacturer’s instructions. The corrected data for day 13 (time 0) was compared to data from day 14 (time 1), and the values were also standardized according to the cell number per insert. Finally, data were normalized to the production rate of the LT control cell line.

### 2.6 Transmission electron microscopy

Cells on inserts were fixed in 2.5% glutaraldehyde in phosphate buffer (0.1 M, pH = 7.2) for a minimum of 4 h at 4°C. The samples were then washed in phosphate buffer (0.1 M, pH = 7.2), postfixed in 1% osmium tetroxide, dehydrated in graded ethanol, and embedded in an epon/araldite resin mixture and polymerized at 60°C for at least 16 h. Semithin sections of 1 μm thickness were cut on a Leica Reichert ultra-microtome UCT (Vienna, Austria), stained with toluidine blue and examined using a light microscope to locate kidney glomeruli. Silver/gold interference color ultrathin sections were cut and mounted on 200 mesh copper grids, contrasted with uranyl acetate and lead citrate, before being examined in a JEM-JEOL1010 transmission electron microscope (JEOL, Tokyo, Japan).

### 2.7 Sample preparation and proteomics

#### 2.7.1 Sample collection

For preservation, cells were collected and washed twice with PBS. Following centrifugation at 4°C at 14,000 × g for 25 min, cells were snap frozen in liquid nitrogen and stored at −80°C until further analysis.

For each sample, at least 1 million cells were solubilized in RIPA lysis buffer (0.1% sodium dodecyl sulphate -SDS-, 0.25% Na-deoxycholate, PBS) containing complete Mini EDTA-free proteinase inhibitors (Roche). Following overnight acetone precipitation, protein pellets were stored at −20°C until trypsin digestion and peptide purification.

#### 2.7.2 In-solution trypsin digestion of proteins

About 200 μg of proteins digested in protein lysis buffer (0.1% SDS. 0.25% Na-deoxycholate, PBS) were precipitated using ice-cold acetone in 1:8 protein to acetone ratio. The precipitated proteins were dissolved in 8 M urea and reduced and alkylated with 5 mM dithiothreitol for 15 min at 60°C and in 10 mM iodoacetamide for 20 min at room temperature, respectively. Samples were diluted with 50 mM triethylammonium bicarbonate (TEAB), and protein digestion was carried out using Pierce™ Trypsin Protease, MS Grade (1:100) (Thermo Fisher) for 4 h at 37°C, followed by second-step trypsin (1:20) for 12 h. The next day, the peptides were acidified in 1% formic acid followed by clean-up using Sep-Pak C18 material (Waters). For peptide clean-up, we used solvent A (0.1% formic acid) and solvent B (60% acetonitrile in 0.1% formic acid) as the mobile phase. The peptides were passed through the Sep-Pak column, washed with solvent A and eluted from the column using solvent B. The eluted peptides were dried using vacufuge and stored at −20°C until further use.

#### 2.7.3 LC-MS data acquisition

These dried peptides were reconstituted in 20 µL of 0.1% formic acid and 6 µL was injected in duplicate on an Orbitrap Fusion Tribrid mass spectrometer connected with the Dionex UltiMate 3000 nanoflow liquid chromatography system (Thermo Fisher). The peptide mixture was loaded onto an enrichment column (100 μm × 0.5 cm, Nanoviper) at a flow rate of 3 μL/min. Peptides were resolved on an analytical column (75 μm × 50 cm, RSLC C18) at a flow rate of 300 nL/min using a gradient of 5%–35% solvent B (0.1% formic acid in 90% acetonitrile) for 150 min. On mass spectrometer, data were acquired in a data-dependent mode. The precursor MS scans (from m/z 375–1,500) were acquired in the Orbitrap at a resolution of 120,000 (at 200 m/z). The parameters used for the MS1 were the automatic gain control (AGC) target 3 × 10^5^ and ion filling time set at 100 ms. The most abundant ions with charge state 2–7 were isolated in 2-s cycles and fragmented using high energy collision dissociation fragmentation with 35% normalized collision energy and detected on Ion Trap at rapid scan rate. The parameters for MS/MS were the AGC target set as 1 × 10^4^ and ion filling time set at 50 ms, with dynamic exclusion set for 30 s and a 10- parts per million (ppm) mass window.

#### 2.7.4 LC-MS data processing

The MS/MS database searches were carried out using Mascot server (version 2.5.0; Matrix Science Ltd., London, United Kingdom), through Proteome Discoverer platform (version 2.2; Thermo Scientific) against the *Homo sapiens* UniProt database, version 2021, containing 564,918 protein sequences. Trypsin was specified as the protease and a maximum of two missed cleavages were allowed. The search parameters involved carbamidomethylation at cysteine which was set as the fixed modification, while oxidation of methionine was set as a variable modification. MS and MS/MS mass tolerances were set to 10 ppm and 0.5 Dalton (Da), respectively. Only Master Proteins (containing at least one unique peptide, and ≥2 PSMs) and 95% confidence interval threshold (*p* < 0.05, Mascot score verification). The mass spectrometry data generated from this study have been deposited in the ProteomeXchange Consortium (http://www.proteomexchange.org) *via* the PRIDE partner repository, with the dataset identifier PXD039214.

## 3 Statistics

The numeric data was normalized to the control LT cell line values, or otherwise as stated in the respective figure legends. The statistical package for social science (SPSS) or GraphPad Prism 9.0 statistic software were used for graphic representation as well as two tailed t-test statistics comparison, *n* ≥ 3. For proteomics, the data is “high quality” when *q* ≤ 0.05, of three cell lines and three independent experiments.

## 4 Results

### 4.1 hiPSCs differentiation into podocytes

**FIGURE 1:**
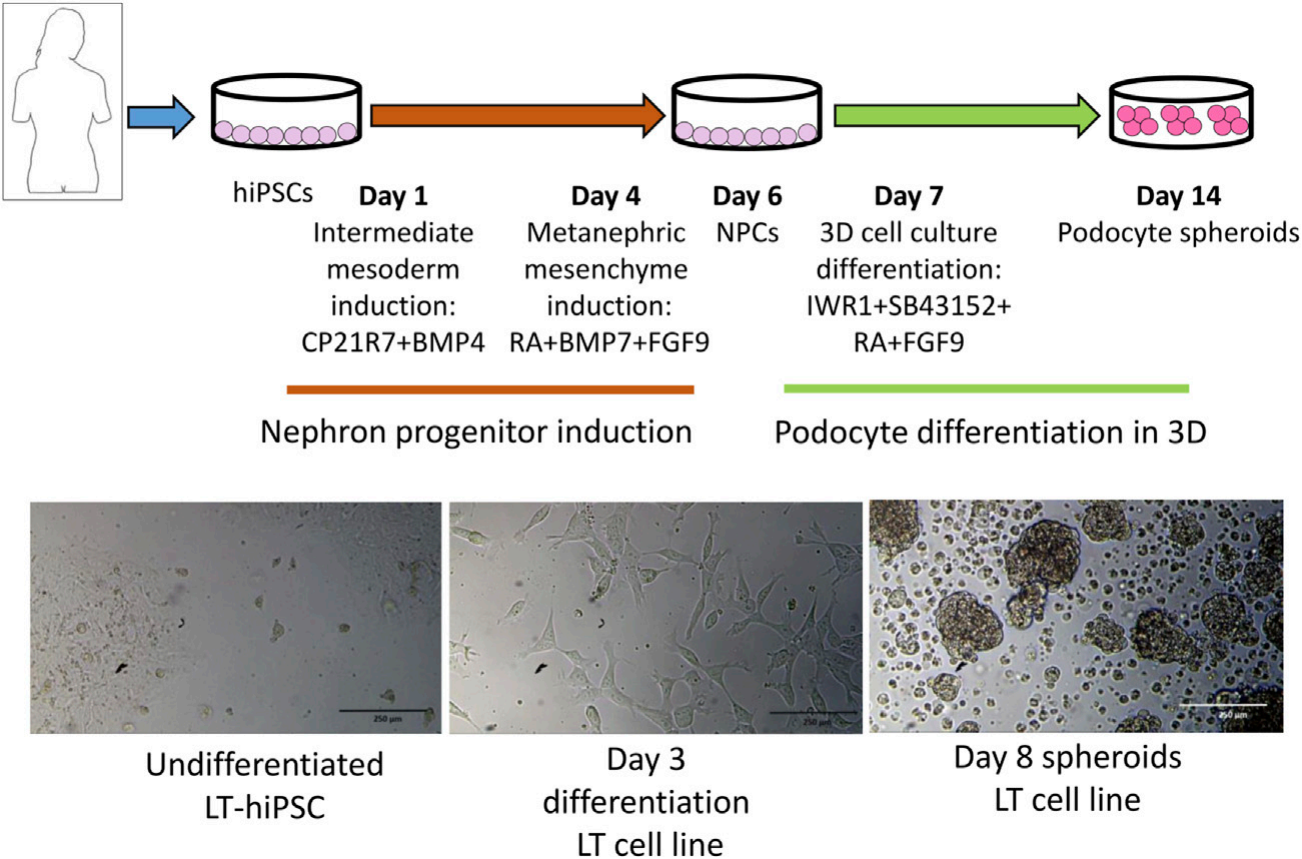
Protocol outline highlighting the different stages and induction media used to induce intermediate mesoderm, nephron progenitor cells (NPCs) and podocytes. At the bottom are representative images of different differentiation stages.

**FIGURE 2:**
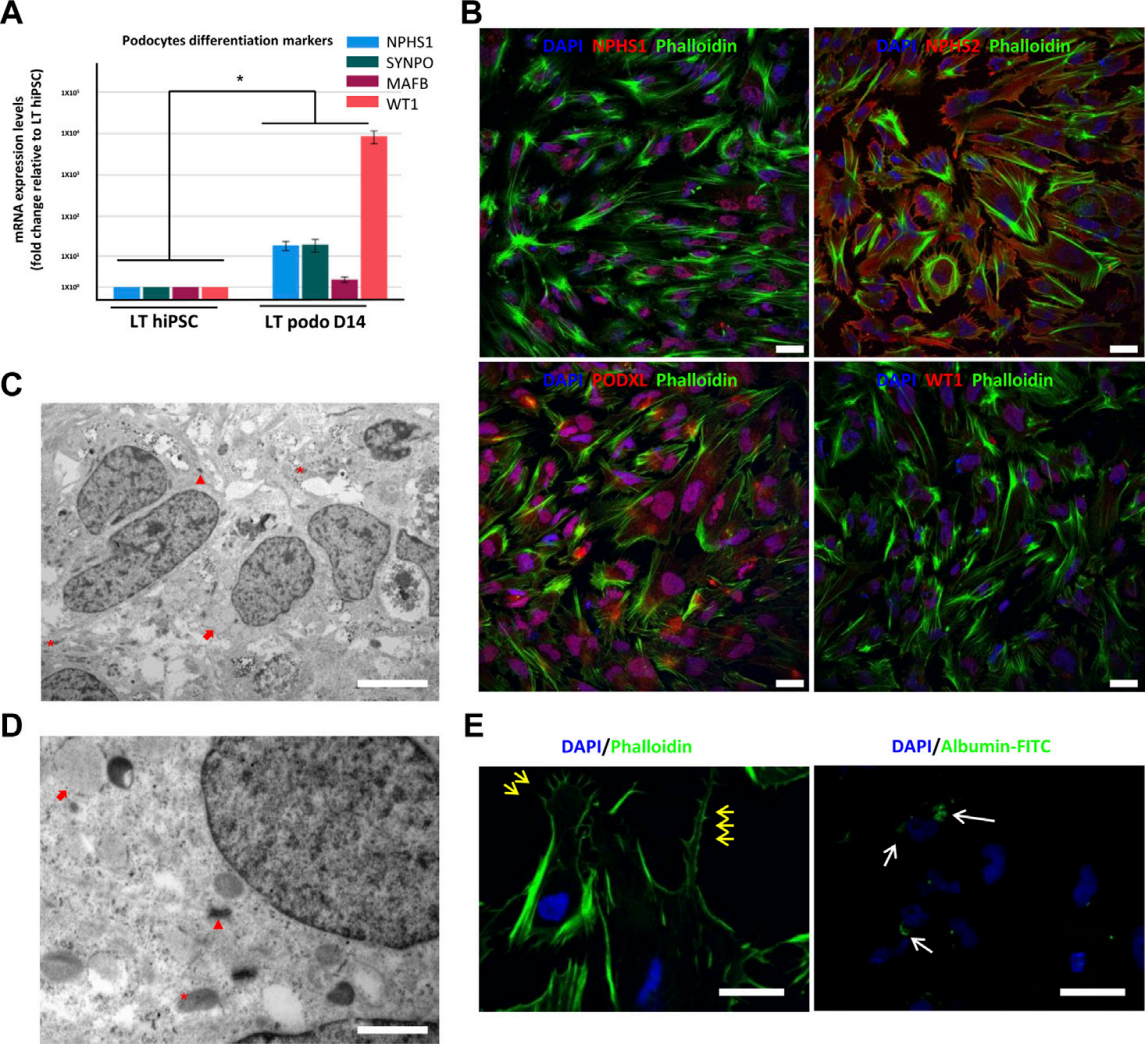
Characterization of hiPSC-derived podocytes. hiPSCs differentiated for 14 days using our induction protocol generate podocytes expressing mRNA of the podocyte markers NPHS1, SYNPO, MAFB, and WT1 **(A)**. In **(B)**, immunofluorescence of NPHS2 (podocin), NPHS1 (nephrin) and WT1 in LT hiPSC-derived D14 podocytes. Scale bar 20 μm. **(C)** and **(D)** are electron micrographs of day 14 podocytes highlighting the mitochondria (asterisk), well developed desmosomes (arrowhead) and developing RER (arrow), magnification 5,000X, scale bar 2,000 nm **(C)** 200,00X, scale bar 500 nm **(D)**. In **(E)**, primary and secondary foot process (yellow arrows) evidenced by phalloidin staining, and on the right image endocytosis of FITC conjugated albumin by LT day 14 podocytes. Scale bar 30 μm. Significance was tested by an unpaired *t*-test. Asterisk denotes statistically significant differences compared to undifferentiated LT hiPSC,*p < 0.05, *n* = 8.

Previous studies have shown that the 3D tissue environment is crucial for the differentiation and phenotypical maturation of iPSC-derived podocytes (Taguchi et al., 2014; Takasato et al., 2014; 2015; Taguchi and Nishinakamura, 2017). However, the costs and complexity of existing protocols (e.g., use of growth factors and need for purification of nephron progenitor cells), as well as the presence of off-target cells in organoids have hindered the broader adoption and use of these methods in modeling podocytopathies *in vitro*. Here, we set out to develop a simple and robust protocol for the generation of 3D human podocytes *in vitro*, by combining a highly efficient and fast 2D protocol for generating nephron progenitor cells (NPCs) (Ciampi et al., 2016) with kidney organoid cultures (Yoshimura et al., 2019) (Figure 1). First, hiPSCs were exposed to the WNT agonist (CP21R7) and BMP4 to induce intermediate mesoderm in 3 days, followed by RA, BMP7 and FGF9 treatment to induce NPCs. At day 7, differentiating cells were gathered and transferred to cell inserts, and treated with the WNT antagonist IWR1, the transforming growth factor-β/small mother against decapentaplegic (TGF-β/Smad) signaling inhibitor SB43152, RA, and FGF9 for an additional 8 days. The cells differentiated further and grew into podocyte 3D spheroids that strongly expressed key podocyte markers such as Nephrin, Wilms tumor-1 (WT1), maf musculoaponeurotic fibrosarcoma oncogene homolog B (MAFB), and Synaptopodin (SYNPO), as determined by using mRNA Q-PCR relative expression and immunofluorescence analyses (Figures 2A, B). Moreover, some features of maturing podocytes, such as membrane folds, desmosomes, and mature rough endoplasmic reticulum (RER) were detected (Figures 2C, D). Podocytes dissociated from spheroids, exhibited mature features of podocytes, such as primary and secondary foot processes and the ability for albumin uptake (Figure 2E). Together, these data show that our combined protocol can simply and efficiently yield human adult-like podocytes.

### 4.2 Proteomics discovery of signaling pathways altered in AS

**FIGURE 3:**
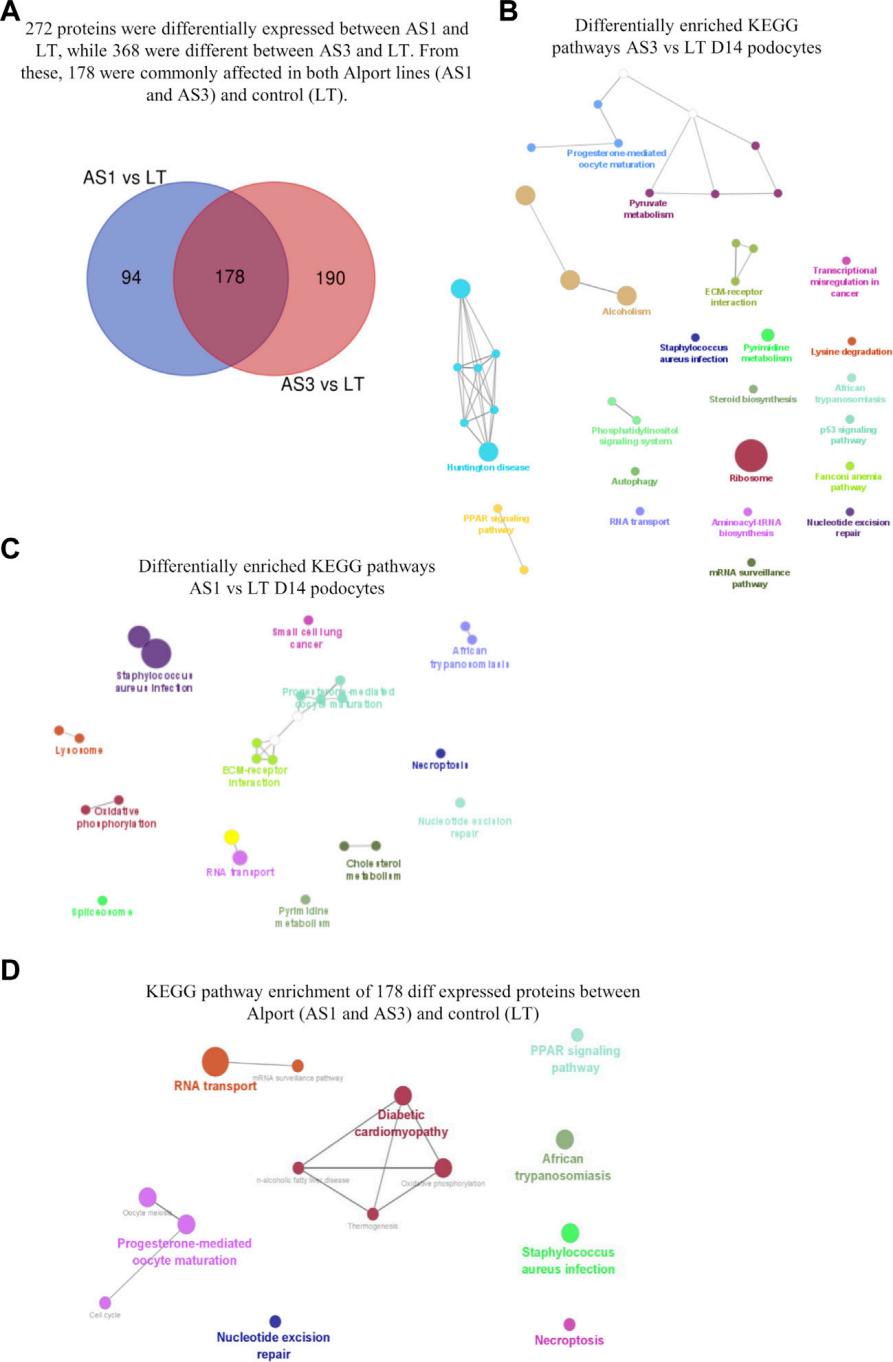
Proteomic analysis comparison of hiPSC-derived D14 podocytes from Alport disease (AS1 and AS3) and normal patients (LT) cell lines. In **(A)**, the Venn diagram highlighting the differentially expressed proteins from each comparison and those commonly affected in both Alport cell lines. The enriched KEGG pathways identified from the differentially expressed proteins between AS3 and LT **(B)**, AS1 and LT **(C)** and Both AS1 and AS3 vs. LT **(D)**. The analysis was performed using Cytoscape v 3.9.1 and its plug-in Cluego using the set of proteins differentially expressed (*p* < 0.05) referred in each comparison.

Next, we used our differentiation method to generate podocytes using hiPSC lines that were derived from patients with AS and compared them with healthy donor-derived podocytes by using discovery proteomics. Importantly, the two Alport cell lines differed in genotype and the mutated collagen: in the AS1 cell line, the mutation was COL4A3 c.345delG; p.[P116Lfs*37], while in the AS3 cell line, the mutation was COL4A5 c.4034G>A; (G1345D). This difference was essential to help us to assess whether, at the molecular level, there were common pathways activated in AS, regardless of the type of mutation and collagen α-chain affected. Proteomic analysis detected over 4,000 proteins from each cell line, and a comparison between the protein levels in Alport podocytes and healthy controls (LT) identified 272 differentially expressed proteins between AS1 and LT podocytes, while 368 proteins were differentially expressed between AS3 and LT podocytes (Figure 3A). A comparison of both panels of proteins indicated that a set of 178 were differentially expressed between Alport (AS1 and AS3) and LT podocytes, regardless of the mutation (Figure 3A). Next, we performed gene ontology (GO) and KEGG pathway analysis using the bioinformatics tools Cytoscape and its plug-in ClueGo (Table 3; Figures 3B, C). The analysis showed that the pathways and GO enrichment were similar for both AS1 vs. LT and AS3 vs. LT comparisons. These GO terms and pathways were then organized in clusters and graphically presented in Figure 3D and Table 4 using ClueGo in Cytoscape. The results were similar when we used the DAVID bioinformatic tool (data not shown). Some of the affected pathways in Alport cell lines that emerged from the GO terms and KEGG pathway enrichment were related to: chromatin organization, RNA maturation and export, OXPHOS, keratinization, ECM-receptor interaction, cell cycle check points, and lipid transport. More detailed analysis of the enriched functional clusters highlighted the common set of proteins affected in different inter-related terms.

**TABLE 3:** GO terms (molecular function, cell compartment, and biological process) most significantly represented (Bonferroni correction), from AS3 to LT comparison, performed with Cytoscape + ClueGo.

ID	Term	Term p-value Corrected with Bonferroni step down	GO Levels	% Associated Genes
*GO:0070013*	intracellular organelle lumen	0.00	[4]	3.30
*GO:0003723*	RNA binding	0.00	[4]	5.10
*GO:0043232*	intracellular non-membrane-bounded organelle	0.00	[4]	3.31
*GO:0005829*	cytosol	0.00	[2, 3, 4]	3.18
*GO:0022626*	cytosolic ribosome	0.00	[3, 4, 5, 6]	18.80
*GO:0045047*	protein targeting to ER	0.00	[6, 7, 8]	18.03
*GO:0000184*	nuclear-transcribed mRNA catabolic process, non-sense-mediated decay	0.00	[7, 8, 9, 10]	16.79
*GO:1903561*	extracellular vesicle	0.00	[5]	4.13
*GO:0044085*	cellular component biogenesis	0.00	[3]	3.54
*GO:0006996*	organelle organization	0.00	[4]	3.29
*GO:0005840*	ribosome	0.00	[5]	10.80
*GO:0044391*	ribosomal subunit	0.00	[3, 6]	12.24
*GO:0010629*	negative regulation of gene expression	0.00	[4, 5, 6]	5.00
*GO:0006364*	rRNA processing	0.00	[6, 7, 8, 9]	9.93
*GO:0034660*	ncRNA metabolic process	0.00	[6, 7]	7.00
*GO:0006413*	translational initiation	0.00	[3, 6, 7, 8]	11.48
*GO:0034470*	ncRNA processing	0.00	[6, 7, 8]	7.56
*GO:0002181*	cytoplasmic translation	0.00	[6, 7, 8]	12.80
*GO:0000956*	nuclear-transcribed mRNA catabolic process	0.00	[6, 7, 8, 9]	10.81
*GO:0042254*	ribosome biogenesis	0.00	[5]	8.47
*GO:0031981*	nuclear lumen	0.00	[5, 6]	3.09
*GO:0005654*	nucleoplasm	0.00	[2, 6, 7]	3.14
*GO:0022613*	ribonucleoprotein complex biogenesis	0.00	[4]	6.85
*GO:0022625*	cytosolic large ribosomal subunit	0.00	[4, 5, 6, 7, 8]	20.97
*GO:0070268*	cornification	0.00	[3, 4, 7, 8, 9]	14.66
*GO:1903561*	extracellular vesicle	0.00	[5]	3.09
*GO:0005882*	intermediate filament	0.00	[6, 7]	7.08
*GO:0043588*	skin development	0.00	[4, 5, 6]	5.06
*GO:0034363*	intermediate-density lipoprotein particle	0.00	[5, 6]	80.00
*GO:0030216*	keratinocyte differentiation	0.00	[5, 6, 7]	5.77
*GO:0034378*	chylomicron assembly	0.00	[4, 5, 8]	38.46
*GO:0045095*	keratin filament	0.00	[7, 8]	10.00
*GO:0062023*	collagen-containing extracellular matrix	0.00	[4, 5]	4.61
*GO:0016584*	nucleosome positioning	0.00	[6, 7, 8, 9]	29.41
*GO:0030261*	chromosome condensation	0.00	[8]	11.43
*GO:0097549*	chromatin organization involved in negative regulation of transcription	0.00	[6, 7, 8, 9, 10, 11, 12]	7.59
*GO:0034371*	chylomicron remodeling	0.00	[5, 6, 9]	36.36
*GO:0099512*	supramolecular fiber	0.00	[4]	3.11
*GO:0008544*	epidermis development	0.00	[4]	4.03
*GO:0070325*	lipoprotein particle receptor binding	0.01	[4]	17.86
*GO:0016607*	nuclear speck	0.01	[3, 4, 8, 9]	4.09
*GO:0042627*	chylomicron	0.01	[4, 5]	25.00
*GO:0016604*	nuclear body	0.01	[2, 3, 7, 8]	3.13
*GO:0071103*	DNA conformation change	0.01	[6]	4.26
*GO:0006397*	mRNA processing	0.01	[6, 7, 8]	3.65
*GO:0031491*	nucleosome binding	0.01	[3]	9.46
*GO:1903047*	mitotic cell cycle process	0.01	[3, 4]	3.04
*GO:0030855*	epithelial cell differentiation	0.01	[4, 5]	3.16
*GO:0050792*	regulation of viral process	0.02	[2, 3]	5.61
*GO:0099513*	polymeric cytoskeletal fiber	0.02	[5, 6]	3.18
*GO:0071827*	plasma lipoprotein particle organization	0.03	[2, 6]	10.34
*GO:0034382*	chylomicron remnant clearance	0.03	[4, 5, 6]	37.50
*GO:0043903*	regulation of biological process involved in symbiotic interaction	0.03	[2, 3]	8.33
*GO:0016458*	gene silencing	0.03	[2, 5, 6, 7]	4.56
*GO:1990204*	oxidoreductase complex	0.04	[3]	6.78
*GO:0045815*	positive regulation of gene expression, epigenetic	0.04	[5, 6, 7]	9.52
*GO:0006997*	nucleus organization	0.04	[5]	5.92
*GO:0034361*	very-low-density lipoprotein particle	0.05	[5, 6]	17.39
*GO:0006323*	DNA packaging	0.05	[7]	4.60

**TABLE 4:** GO terms (molecular function, cell compartment, and biological process) most significantly enriched from the 178 proteins differentially expressed between Alport (AS1 and AS3) and control (LT). Data obtained from Cytoscape + ClueGo, only Bonferroni corrected GO terms with *p* ≤ 0.1) from AS1 and to LT comparison, performed with Cytoscape + ClueGo.

ID	Term	Term p-value corrected with Bonferroni step down	GO Levels	% Associated Genes	# Genes
*GO:0070268*	cornification	0.00	[3, 4, 7, 8, 9]	10.34	12.00
*GO:0030261*	chromosome condensation	0.00	[8]	10.00	7.00
*GO:0031936*	negative regulation of chromatin silencing	0.00	[4, 5, 6, 7, 8, 9, 10, 11, 12, 13, 14, 15]	28.57	4.00
*GO:0005882*	intermediate filament	0.00	[6, 7]	4.87	11.00
*GO:0097549*	chromatin organization involved in negative regulation of transcription	0.00	[6, 7, 8, 9, 10, 11, 12]	6.21	9.00
*GO:0016584*	nucleosome positioning	0.00	[6, 7, 8, 9]	23.53	4.00
*GO:0006323*	DNA packaging	0.00	[7]	4.21	11.00
*GO:0045095*	keratin filament	0.00	[7, 8]	7.00	7.00
*GO:0006997*	nucleus organization	0.00	[5]	5.26	8.00
*GO:0051291*	protein heterooligomerization	0.01	[7]	14.81	4.00
*GO:0045814*	negative regulation of gene expression, epigenetic	0.01	[5, 6, 7]	5.65	7.00
*GO:0048525*	negative regulation of viral process	0.01	[2, 3, 4]	6.45	6.00
*GO:0060968*	regulation of gene silencing	0.02	[3, 4, 5, 6, 7, 8]	4.83	7.00
*GO:0031490*	chromatin DNA binding	0.02	[3, 5]	5.61	6.00
*GO:0034728*	nucleosome organization	0.02	[5, 6, 7]	4.06	8.00
*GO:0070653*	high-density lipoprotein particle receptor binding	0.03	[5]	50.00	2.00
*GO:0010903*	negative regulation of very-low-density lipoprotein particle remodeling	0.03	[3, 4, 5, 6, 7, 8, 10, 11]	50.00	2.00
*GO:0051053*	negative regulation of DNA metabolic process	0.04	[4, 5, 6, 7, 8]	4.35	7.00
*GO:0000077*	DNA damage checkpoint signaling	0.04	[5, 6, 7, 8, 9, 10]	4.24	7.00
*GO:0034363*	intermediate-density lipoprotein particle	0.05	[5, 6]	40.00	2.00
*GO:0000792*	heterochromatin	0.05	[3, 7]	6.10	5.00
*GO:0098803*	respiratory chain complex	0.06	[3, 4]	5.68	5.00
*GO:0004402*	histone acetyltransferase activity	0.07	[8, 9, 10, 11, 12]	4.17	2.00
*GO:0045104*	intermediate filament cytoskeleton organization	0.07	[3, 6]	7.55	4.00
*GO:0005746*	mitochondrial respirasome	0.07	[3, 4, 6, 7, 8, 9]	5.56	5.00
GO:0019646	aerobic electron transport chain	0.08	[4, 6, 7, 8]	5.32	5.00
*GO:0045324*	late endosome to vacuole transport	0.08	[3, 4, 5]	11.54	3.00
*GO:2001033*	negative regulation of double-strand break repair *via* non-homologous end joining	0.08	[6, 7, 8, 9, 10, 11]	28.57	2.00
*GO:0061436*	establishment of skin barrier	0.10	[5, 6, 7, 8]	10.71	3.00
*GO:0006334*	nucleosome assembly	0.10	[6, 7, 8, 9]	4.05	6.00

Overall, the proteomic analysis showed numerous molecular processes and pathways that are affected by Alport, causing alterations in podocytes long before a mature GBM is formed, thus uncovering new potential pathophysiological mechanisms for Alport disease. Moreover, some of the discovered proteins that are related to energy metabolism and cell stress could be of clinical and therapeutic interest since they could be new targets for pharmacological intervention in patients with AS.

### 4.3 Less mature podocyte features in AS cell lines

**FIGURE 4:**
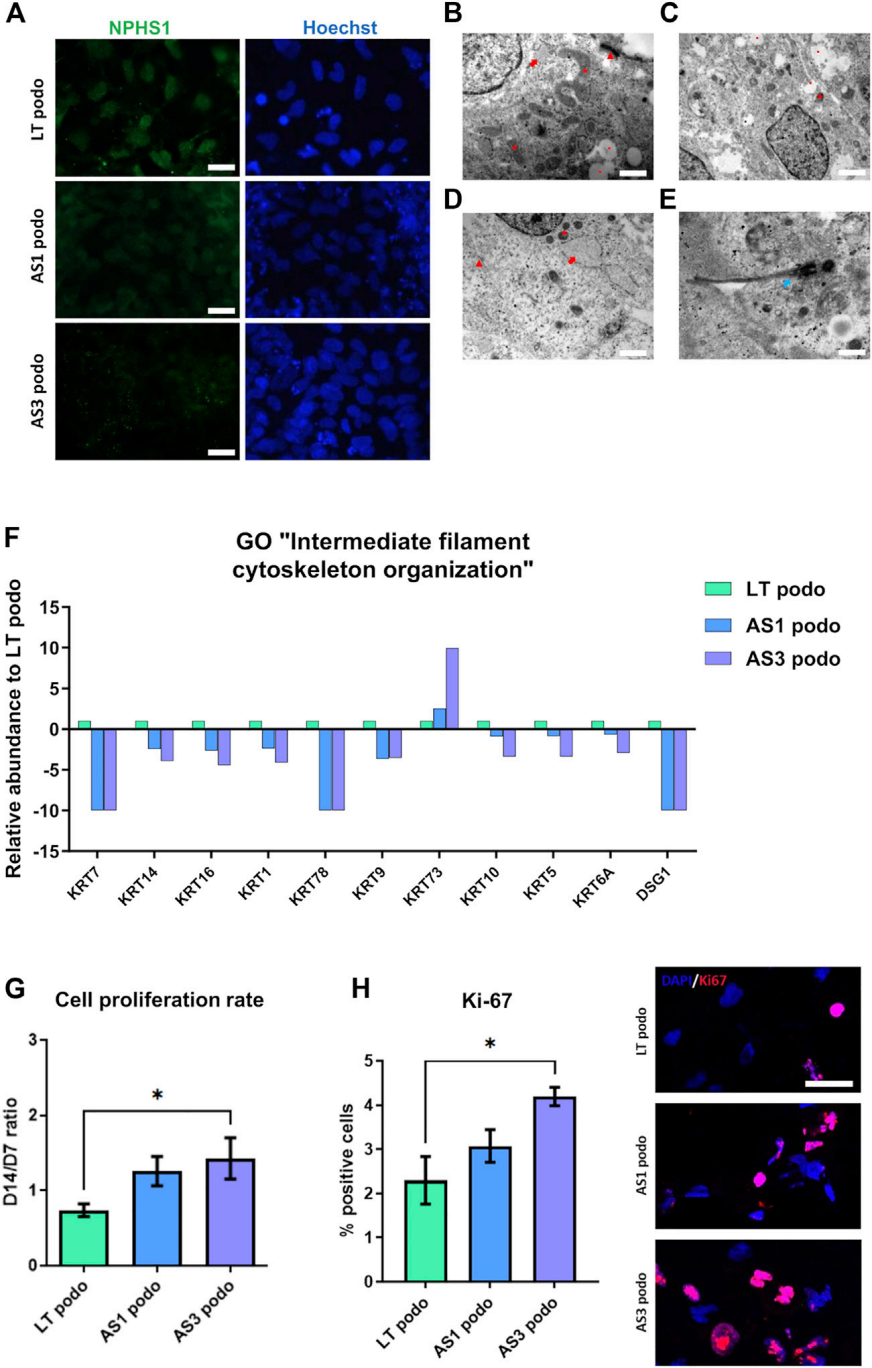
hiPSCs from Alport disease patients were differentiated into podocytes and compared with hiPSC-LT control podocytes. We observed weaker expression of NPHS1 by immunofluorescence in AS3 podocytes compared to AS1 and LT control, scale bar 50 µm **(A)**. **(B)**, electron micrograph of LT-differentiated podocytes showing prominent nucleus, cytoplasm containing large mitochondria (asterisk), developing RER (arrow) with swollen cisternae and mature desmosomes (arrowhead), scale bar 200 nm. **(C,D)**, electron micrographs of AS1 and AS3 podocytes respectively, showing small mitochondria (asterisk), developing and prominent RER (arrow), immature desmosomes (arrowhead) and vacuoles (dot). Respectively, magnification 120,00X, scale bar 400 nm and 25,000 X, scale bar 200 nm. Additionally, features like junctions and desmosomes (red arrows) were less mature in AS1 **(C)** and AS3 **(D)** podocytes compared to LT controls. **(E)**, AS1 podocytes showing a primary cilium (white arrow), 40,000 X, scale bar 125 nm. In **(F)**, several keratins and Desmoglein 1 (DSG1), involved in epithelial maturation and desmosome formation were significantly decreased in Alport cell podocytes compared to LT podocytes control, as shown by the proteomics analysis. **(G)**, Alport cell lines remained in proliferation during day 7 to day 14 spheroid culture. **(H)** Quantification of Ki-67 positive cells normalized over total number of cells and expressed in [%]. Significance was tested by an unpaired *t*-test. Asterisk denotes statistically significant differences compared to AS3 podocytes, *p < 0.05. On the left Ki-67 expression by immunofluorescence. Scale bar 50 µm.

Because the proteomic analysis suggested that the mutations could affect podocyte development, we studied possible phenotypical differences among Alport and control podocytes through imaging techniques. Immunofluorescence analysis showed that Alport podocytes (from both the AS1 and AS3 cell lines) expressed less Nephrin compared to LT control cells (Figure 4A; Supplementary Figure S1). However, we did not see any statistical difference in the mRNA expression of early differentiation markers, such as WT1 or MAFB (Supplementary Figure S1). Interestingly, though, electron microscopy (EM) analysis revealed fewer desmosomes and hemidesmosomes and smaller mitochondria in Alport podocytes, compared to LT control podocytes (Figures 4B–E), further indicating delayed differentiation in Alport podocytes. Additional features in an immature state in some Alport lines were poorly developed RER and primary cilia in some cases (Figure 4E). These data are in line with the poorer epithelial maturation highlighted by the downregulation of various keratins and Desmoglein 1 (DSG1) involved in desmosome and hemidesmosome formation and detected in the proteomic analysis (Figure 4F).

Cell cycle was also one of the pathways that were enriched in GO analysis. Indeed, parallel with the poorer epithelialization of Alport podocytes, we observed that during the maturation stage from NPCs toward podocytes (day 7 to day 14 in spheroids), Alport cells persisted in proliferating, in contrast to LT control podocytes (Figure 4G). This effect was specific to Alport podocytes, since during the induction of NPCs, all cell lines were highly proliferative, without differences between them being observed. Immunostaining for the proliferation marker Ki-67 (Sun and Kaufman, 2018) did indeed reveal higher numbers of Ki-67 nuclei in AS cultures at Day 14 compared to LT ones (Figure 4H), further suggesting persistent mitotic activity in AS lines.

**FIGURE 5:**
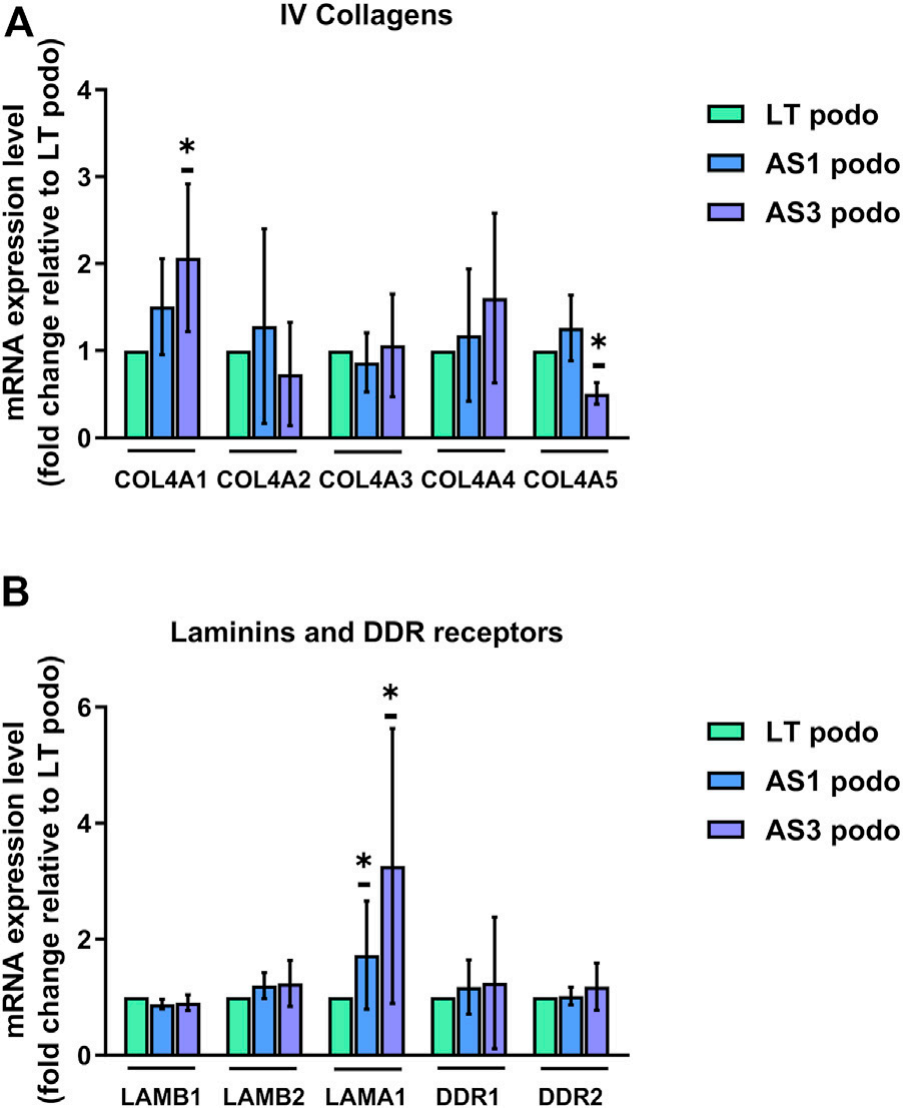
mRNA expression LT vs. Alport podocytes. In **(A)**, mRNA expression of COL4A1, COL4A2, COL4A3, COL4A4, and COL4A5 was analyzed using Q-PCR in Day 14 podocytes from the three cell lines and normalized to the expression level found in LT control podocytes for each experiment. **(B)**, relative mRNA expression of glomerular basement membrane laminins LAMB1, LAMB2, and LAMA1, and collagen receptors DDR1 and DDR2. Significance was tested by an unpaired *t*-test. Asterisk denotes statistically significant differences compared to LT control podocytes,*p < 0.05, *n* = 4.

The expression pattern of type IV collagens was consistent with the delayed maturation in Alport podocytes. In particular, we observed a decreased expression of COL4A5 in the AS3 compared to LT control podocytes, and a simultaneous increase in the expression of COL4A1, the predominantly fetal collagen isoform of GBM (Figure 5A). A similar tendency in COL4A1 was observed in AS1, although there was no significant difference. No significant changes were observed in COLA2, A3, and 4, either). In addition, there was a significant upregulation of laminin α 1 (LAMA1) in Alport podocytes, another ECM protein associated with immature podocytes, while another laminin, laminin β 2 (LAMB2) also tended to be upregulated in mutant cell lines (Figure 5B). Altogether, these results show that AS1 and AS3 are less mature than LT control podocytes, implying that collagens play a signaling role in podocyte differentiation and cell cycle progression, and not only as structural components of the GBM. As a matter of fact, several integrins and transmembrane proteins that interact with laminins and collagens and play signaling roles in many cell types, were detected in the proteomic analysis, while the collagen-specific receptors (Discoidin domain receptor) DDR1 and DDR2 were strongly upregulated in differentiated podocytes compared to undifferentiated hiPSCs (Supplementary Figure S2). Together, these findings imply that collagens act as signaling ligands for podocyte differentiation and maturation.

### 4.4 Energy metabolism is dysregulated in alport podocytes

**FIGURE 6:**
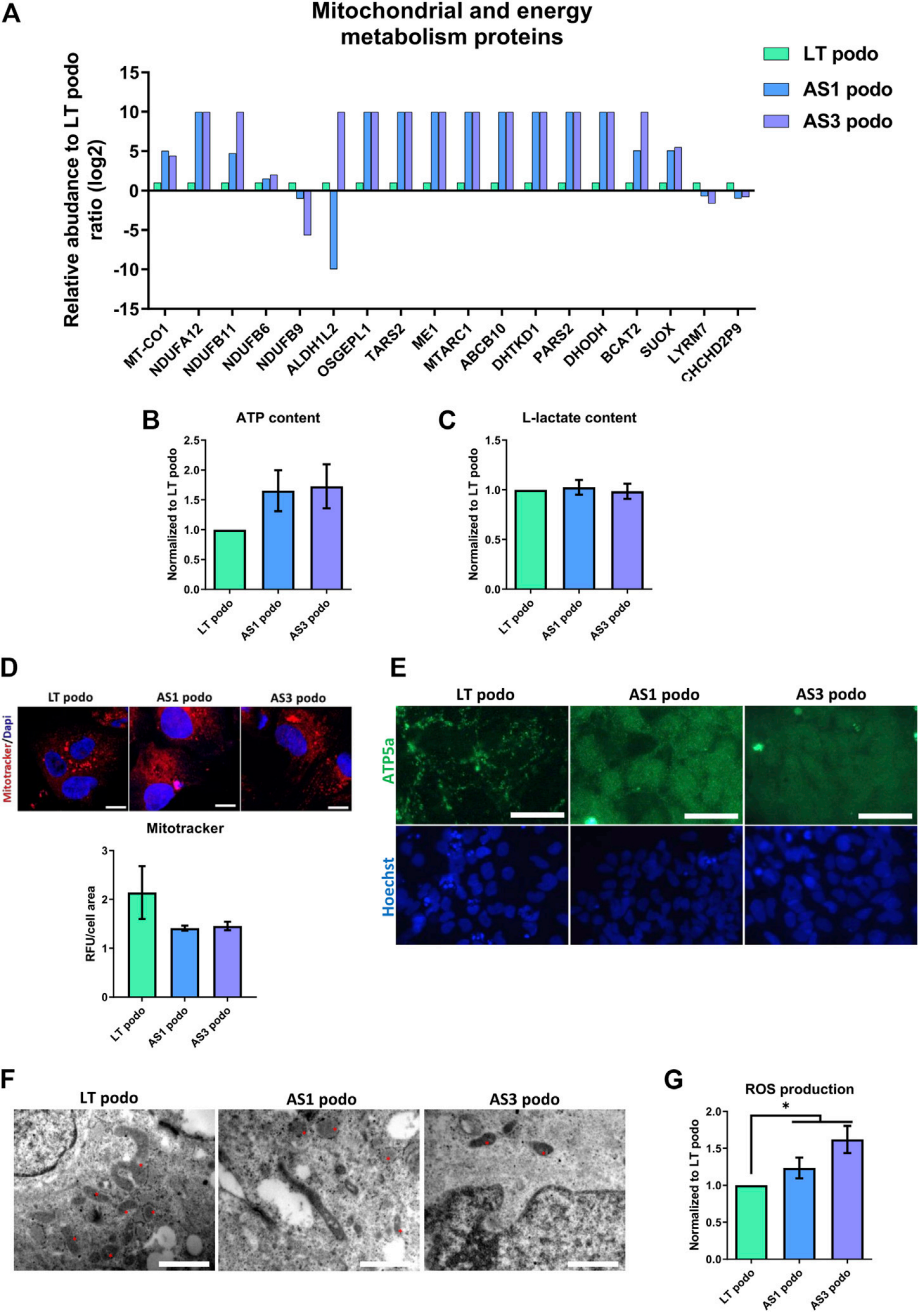
Metabolism in Alport Day 14 podocytes. In **(A)**, several proteins involved in mitochondrial metabolism were dysregulated in Alport cell lines. No statistical difference was observed in ATP content **(B)**, L-Lactate production **(C)** or mitochondrial membrane potential (IF, scale bar 20 μm, the MitoTracker fluorescent intensity was normalized over cells area for quantification) **(D)**. However, mitochondrial morphology varied among the cell lines, as demonstrated by ATPase IF, scale bar 50 µm **(E)**, and electron microscopy **(F)**. In EM the morphology of mitochondria (asterisks) in the different cell lines is highlighted; magnification 250,00X for LT podo and 200,00x for AS podo figure, scale bar respectively 400 nm and 500 nm. Alport cell lines produced more reactive oxygen species than LT control podocytes **(G)**. Significance was tested by an unpaired *t*-test. Asterisk denotes statistically significant differences compared to LT control podocytes,*p < 0.05, *n* = 9.

Based on the differences between metabolic pathways of AS and control podocytes that emerged from the proteomic analysis, and the observation that several mitochondrial proteins, including those of respiratory complexes I, III and IV were altered (Figure 6A), we evaluated the energy balance in the cells in terms of ATP content, mitochondrial function and glycolysis.

The data obtained indicated that in spite of a decrease in ATP content in Alport podocytes, there was no statistical difference between the different groups (Figure 6B). ATP can be produced using two main pathways: glycolysis in the cytoplasm and oxidative phosphorylation in the mitochondria. In order to evaluate if there was a difference in the contribution to the energy metabolism between cell types, L-Lactate production—a byproduct of glycolysis that is actively transported out of the cell—was measured in the supernatant of conditioned media by control (LT) and Alport (AS1 and AS3) podocytes using a colorimetric assay. The results showed again that there was no difference in the level of L-lactate production between the different podocyte groups either (Figure 6C). Parallel to this, we studied mitochondrial functionality by measuring membrane potential with the Mitotracker probe and JC-1 assay. Again, the results indicated that there was no statistical difference in membrane potential across cell lines (Figure 6D; Supplementary Figure S3). Together, these assays indicate that there are no energy deficits in Alport cells compared to controls.

However, due to the fact that several mitochondrial proteins were differentially expressed in Alport podocytes compared to controls, one could assume that although the energy production seems normal, the fitness and/or functionality of this organelle is altered in AS. In fact, there was a marked difference between the ATP5A—a mitochondrial ATP synthase that catalyzes ATP synthesis, found in LT—and Alport podocytes. In the former, a net-like punctuated pattern was observed, while Alport podocytes exhibited a diffuse signal throughout the cell cytoplasm (Figure 6E), which is indicative of less mature mitochondria (Varum et al., 2011; Noguchi and Kasahara, 2018) (Figure 6E). In addition, EM showed that mitochondria in LT podocytes were bigger and more connected than those observed in AS1 and AS3, which were mostly small, rounded, vacuolated and rarely fused or in contact with each other (Figure 6F). Growing podocytes with such mitochondria patterns could in principle perform with biophysically suboptimal metabolism and size, and a permanently inefficient homeostatic status (Lavecchia et al., 2022). The morphological difference between mitochondria and the altered expression of several metabolism-associated proteins could imply higher production of reactive oxygen species (ROS) and cellular stress. Indeed, our analysis showed a higher production of ROS in AS1 and AS3 podocytes than in LT control ones (Figure 6G), indicating that the pathobiology of Alport disease may involve an imperfect mitochondrial metabolism that contributes to oxidative stress. In line with these results, PPARα and PPARγ mRNA, which both are crucially involved in ROS generation, peroxysome homeostasis and cell proliferation (Schrader and Fahimi, 2006), were also upregulated in Alport podocytes (Supplementary Figure S4).

Together these results highlight that Alport mutations in COL4A3 and COL4A5 lead to alterations in mitochondrial function, increased cellular stress and increased production of ROS.

## 5 Discussion

### 5.1 Disrupted pathways in human AS

In this work, our cell culture model and proteomic analysis enabled us to uncover hitherto unknown pathways and biological mechanisms that contribute to the pathobiology of AS. In particular, our results show that Alport-causing mutations affect several metabolic pathways and delay the maturation of podocytes in 3D culture.

AS is considered to be caused by changes in the physical composition of the GBM that lead to early dysfunctional manifestations, such as hematuria. However, there is no clear understanding of the mechanisms that govern AS pathogenesis, and whether there are any phenotypical or molecular alterations in podocytes during development or before the first clinical signs appear. Here, we revealed simultaneous alterations in the mitochondrial metabolism, epithelial maturation, lipid metabolism, cell cycle, and RNA maturation (among others) in patient-derived podocytes. As such, these findings add a key piece of the puzzle of AS pathophysiology, by demonstrating that metabolic disturbances appear in Alport podocytes before the formation of a functional GBM. This could imply that some of the etiopathogenic pathways could act “silently” before the disease onset. Importantly, many of these pathways can potentially be pharmacologically targeted. For example, in this study we observed changes in PPAR expression between Alport and control podocytes, which has been thoroughly investigated in other kidney disease models (Kiss-Tóth and Roszer, 2008) and has been pharmacologically targeted with promising results (Yang et al., 2006) (reviewed in Romero-Guevara et al., 2020).

AS is characterized by an incomplete switch from fetal α1 α1 α2 to mature α3 α4 α5 collagen type IV trimers in the basement membrane, which indicates the existence of a quality check point during collagen assembly. If mutations impair the trimerization of mature α chains, the GBM maintains its fetal constitution, rather than an array of mutated mature collagens. How this shift takes place is not known, but integrins and DDR collagen receptors could be involved in this switch, as they can be activated by single α chains without these being previously assembled in a trimeric structure, as previously thought(Yeh et al., 2012; Kim et al., 2020). In this sense, through proteomics and molecular analysis, we detected several collagens and integrins, as well as collagen receptors that could be part of a regulatory loop controlling the development progression of podocytes *in vitro.* Moreover, our Q-PCR analysis showed that the point mutation in COL4A5 in the AS3 cell line was associated with a downregulation of this gene, and simultaneous upregulation of COL4A1 and LAMA1, supporting the idea that gene expression of the different collagen α chains is tightly controlled by quality check points. Moreover, the low expression of COL4A5 in the AS3 cell line may be due to excessive instability of the mutant mRNA (Roos and de Boer, 2021; Wu et al., 2022).

### 5.2 Cell cycle and delayed differentiation by Alport mutations

Our data supports the hypothesis that Alport mutations caused the delayed or incomplete differentiation of podocytes. Alport podocytes exhibited less mature features compared to the control LT cell line, such as lower expression of nephrin, less mature desmosomes, junctions and smaller mitochondria. An important difference was the proliferation rate of the cells, which highlighted that Alport cell lines continued to divide during 3D culture, while no further proliferation was observed in the controls. This result suggests that AS1 and AS3 remained in a more progenitor-like stage, while control cells arrested proliferation and continued their maturation process. Approximately two-fold difference in cell numbers between Alport lines and the LT control was observed. This is consistent with the upregulation of several cell cycle proteins in Alport cell lines. In addition, keratins, cytoskeletal proteins involved in epithelial maturation, were markedly downregulated in AS1 and AS3, providing additional evidence that Alport podocyte maturation was hampered.

### 5.3 ROS and metabolic stress in Alport-derived podocytes

Mouse and immortalized cell line models of AS have shown that ER stress (measured as activation of unfolded protein response pathway), as well as metabolic alterations, occur in AS (Pieri et al., 2014; Jao et al., 2019). Other studies in AS mice reported defective mitochondrial respiration and disrupted mitochondrial morphology in isolated tubular cells as well as alterations in mitochondrial homeostasis very early in the disease development (Ding et al., 2018; Nicolaou et al., 2020). In this study, we found several pieces of evidence that highlight metabolic disturbances in mitochondrial proteins, possibly due to excessive ROS production and delayed maturation in AS patient-derived podocytes.

ROS are by-products of mitochondrial respiration, which can cause oxidative stress and contribute to pathophysiological processes at the cellular level that can lead to cell death and/or autophagy, by means of mitochondrial dysfunction and DNA and protein damage. A ROS increase, on the other hand, can be due to ER stress or lipotoxicity, which has been observed in other Alport models [reviewed in (Tharaux and Huber, 2012)]. While ROS production is a physiological process, in excessive amounts it can have a detrimental effect on the cell. In our setting, the higher production of ROS by Alport podocytes was associated with the altered expression of several mitochondrial proteins, and at the microscopic level, a more immature mitochondrial morphology in Alport cells.

Although it is unclear how the observed metabolic alterations occur from a mechanistic point of view, we can infer that the mutated collagens caused a delay in the maturation process and activated or maintained the proliferation of Alport podocytes, which eventually led to persistently higher energy demands and oxidative stress. In addition to these biological changes, mutations could have caused additional stress in other pathways shown to be affected in Alport. ER and lipid stress may have contributed to the excess production of ROS, which through a positive feedback loop can further induce proliferation and possibly affect the viability of podocytes in the long term. Indeed, we observed the upregulation of apolipoproteins in Alport podocytes and known pathways that affect podocytes in other disease models, which have been connected to lipotoxicity. The DNA check point pathway that was enriched in the GO analysis can also be explained (at least partially) by the excess of ROS, which causes DNA damage.

## 6 Conclusion

The analysis of patient-derived podocytes provides evidence that collagen mutations, apart from contributing to the defective development of the glomerular filtration barrier, can cause significant alterations in podocyte development and metabolism very early during development, possibly even before the formation of the filtration barrier. Apart from the new knowledge of the pathogenesis and pathways affected in AS, our study provides a new platform for studying AS using human podocytes derived from patient hiPSCs. One limitation of this study is that the podocytes are studied in isolation while in reality the GBM co-exists with podocytes, endothelial cells and the tissue microenvironment. Nonetheless, our method yielded podocytes with well-defined primary and secondary foot processes as well as endocytic activity, characteristics of a high morphological maturation level, which had hitherto been observed primarily *in vivo* (Russo et al., 2007; Grgic et al., 2012) or after transplantation of fetal kidney rudimental tissues (Xinaris et al., 2012; Xinaris et al. 2016).

This is an advantage compared to other models that use human immortalized podocyte cell lines or animal settings, since hiPSCs share more physiological characteristics with endogenous podocytes, as well as providing increased plasticity for organization into more complex structures, such as organoids. Another advantage is the fact that the commercially available hiPSCs are directly derived from AS patients and are therefore clinically relevant genotypes. In this regard, the majority of Alport mouse models are KO or transgenic, alterations which rarely occur in patients. Our platform could be further optimized and used to study a wide range of genotypes and potentially establish genotype-phenotype correlations using specific cellular features in hiPSC-derived models.

## Data Availability

The mass spectrometry data generated from this study have been deposited in the ProteomeXchange Consortium (http://www.proteomexchange.org) *via* the PRIDE partner repository, with the dataset identifier PXD039214.

## Supplementary material

SUPPLEMENTARY FIGURE S1
**(A)** Nephrin immunofluorescence signal quantification. (Significance was tested by an unpaired *t*-test. Asterisk denotes statistically significant differences compared to LT control podocytes, **p*<0.05). **(B)** mRNA relative expression of WT1 and MAFB was analyzed by QPCR in Day 14 podocytes of the three cell lines and normalized to the expression level found in LT control podocytes for each experiment. Significance was tested by one-way ANOVA followed by Bonferroni test, *n* = 3.

SUPPLEMENTARY FIGURE S2 mRNA relative expression of DDR1 and DDR2 was analyzed by QPCR in Day14 podocytes of the three cell lines and normalized to the expression level found in undifferentiated LT hiPSCs control. Significance was tested by an unpaired *t*-test. Asterisk denotes statistically significant differences compared to LT control podocytes, **p* < 0.05, *n* = 4.

SUPPLEMENTARY FIGURE S3 J-1 expression by immunofluorescence (scale bar 20 µm) and quantification of JC-1 red to green fluorescence intensity ratio (i.e., aggregate to monomer ratio) (Significance was tested by an unpaired *t*-test).

SUPPLEMENTARY FIGURE S4 mRNA relative expression of PPARα and PPARϒ was analyzed by QPCR in Day14 podocytes of the three cell lines and normalized to the expression level found in LT control podocytes for each experiment. Significance was tested by one-way ANOVA followed by Bonferroni test. Asterisk denotes statistically significant differences compared to undifferentiated LT hiPSC, *p < 0.05, *n* = 3.
