# Supplementary figures and images for: Patient-derived podocyte spheroids reveal new insights into the etiopathogenesis of Alport syndrome
Source: Front Cell Dev Biol. 2023 Mar 2;11:1111424. doi: 10.3389/fcell.2023.1111424 (PMC10018139; doi:10.3389/fcell.2023.1111424)

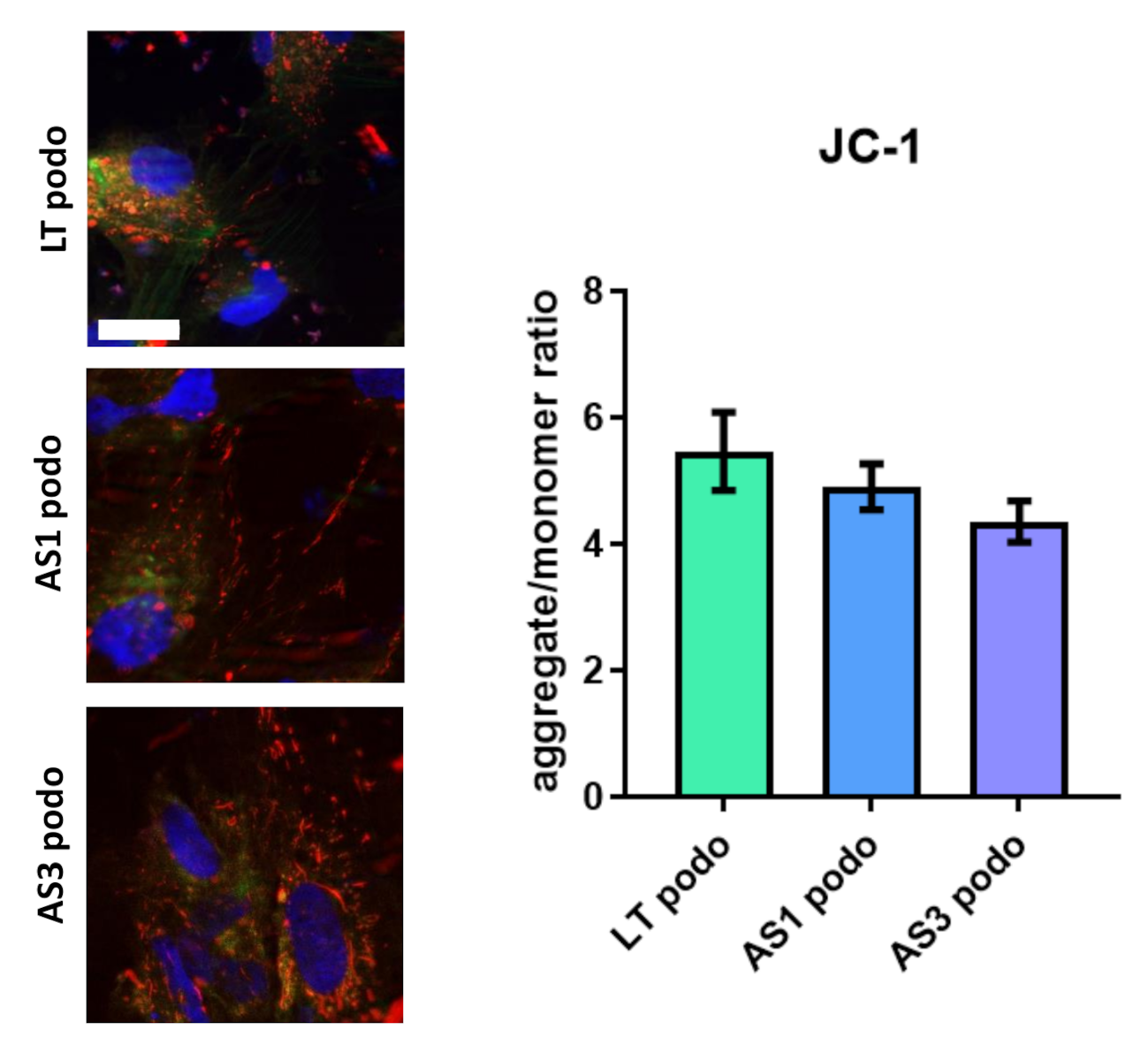

Supplement: Supplementary file 1 [file Image3.TIF]

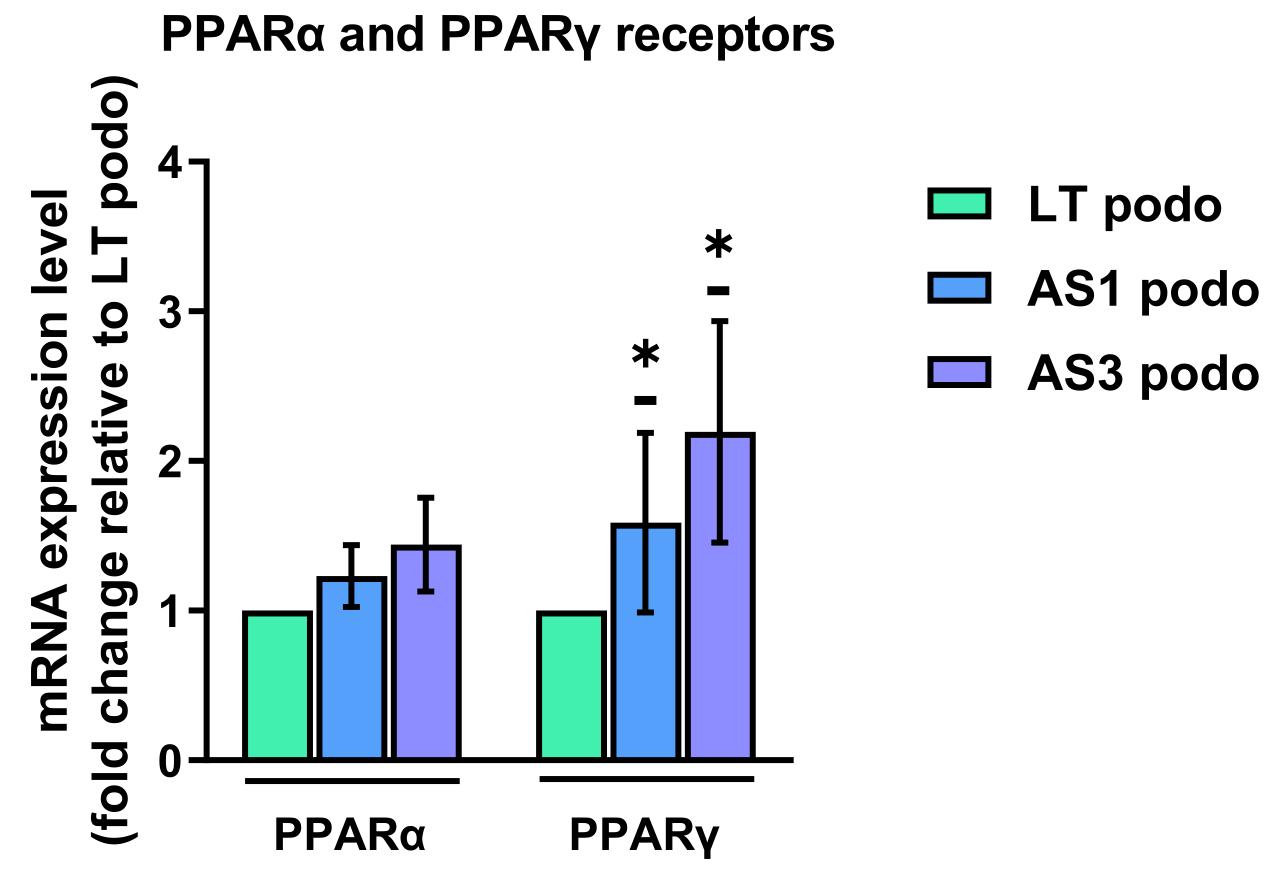

Supplement: Supplementary file 2 [file Image4.TIF]

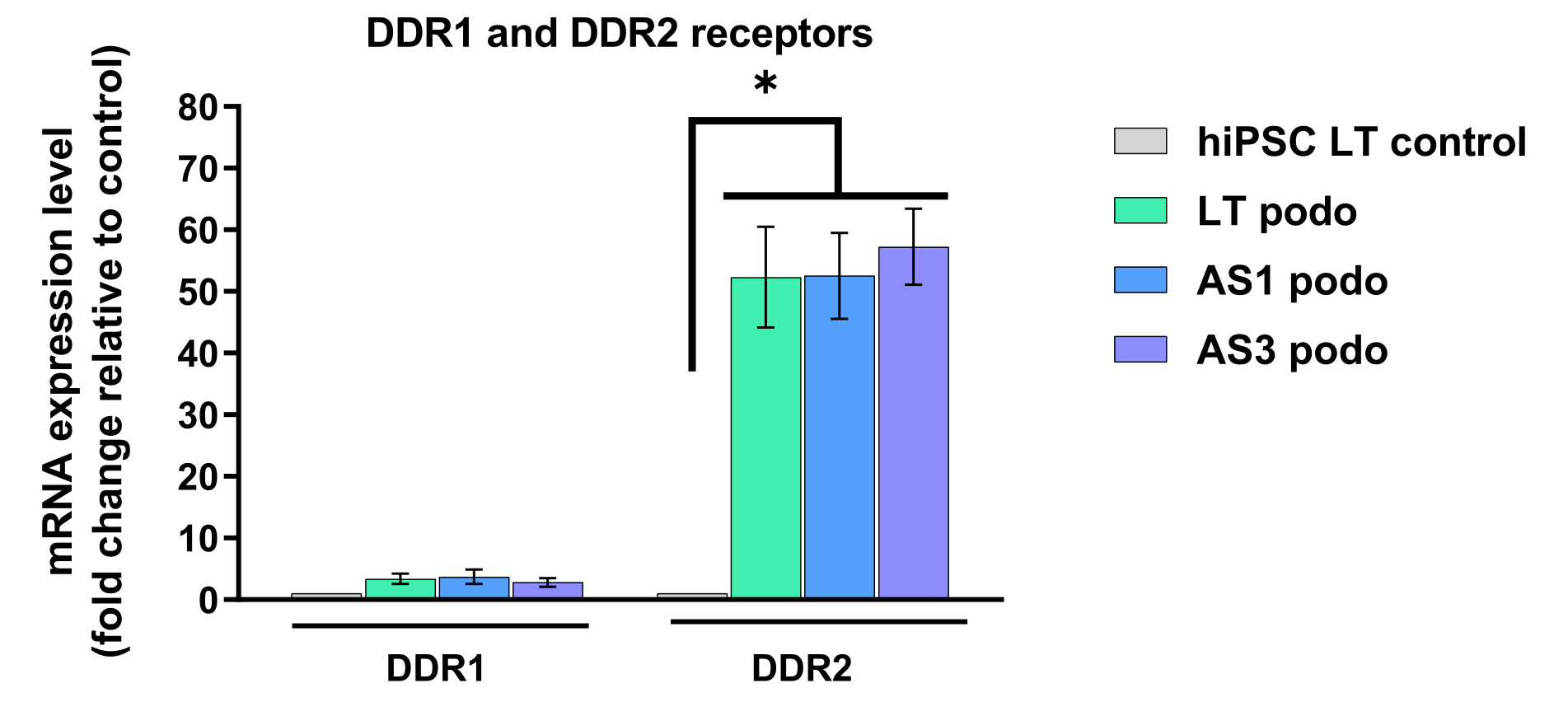

Supplement: Supplementary file 3 [file Image2.TIF]

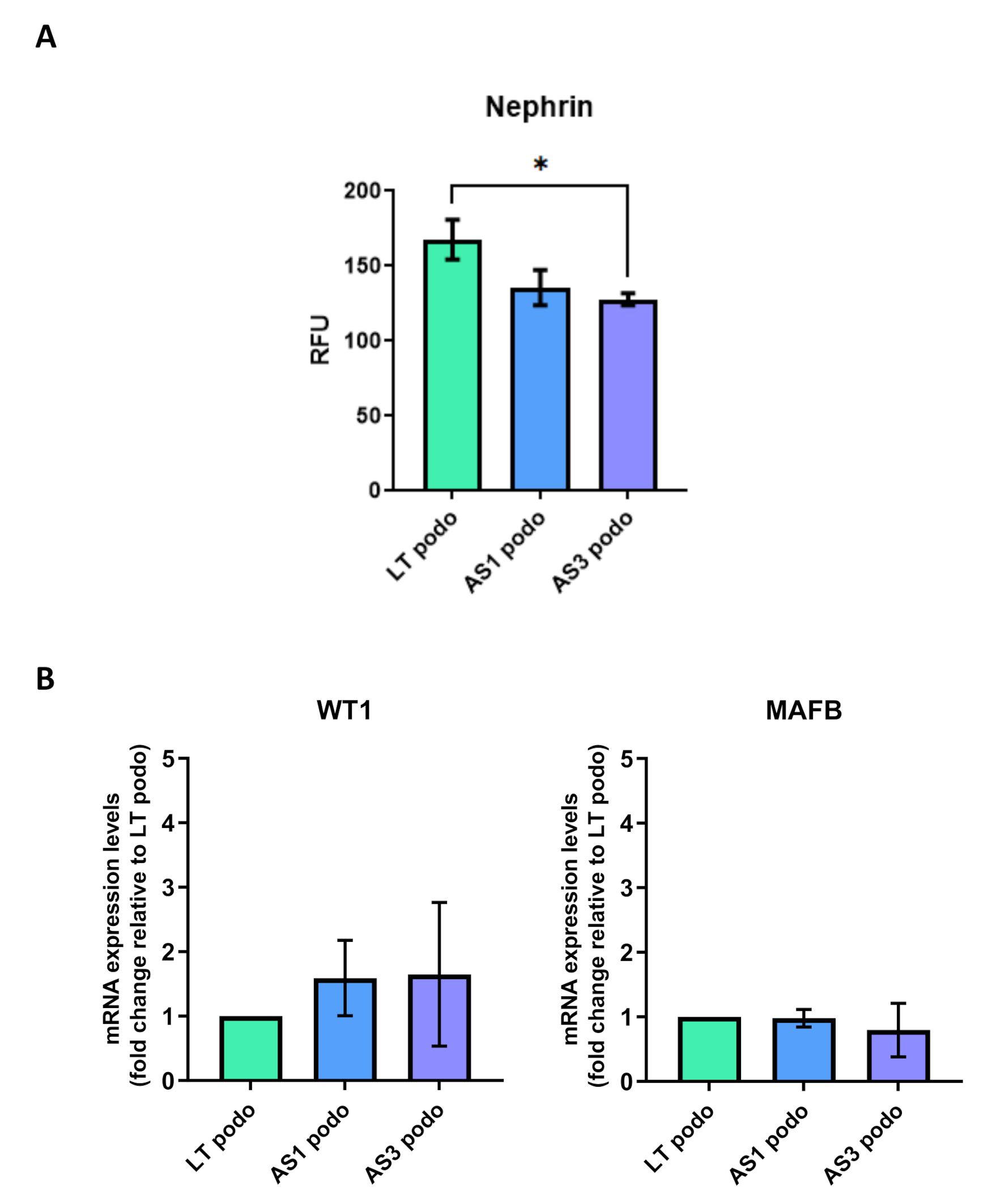

Supplement: Supplementary file 4 [file Image1.TIF]
